# Nanoparticles—Emerging Potential for Managing Leukemia and Lymphoma

**DOI:** 10.3389/fbioe.2017.00079

**Published:** 2017-12-18

**Authors:** Raquel Vinhas, Rita Mendes, Alexandra R. Fernandes, Pedro V. Baptista

**Affiliations:** ^1^UCIBIO, Departamento de Ciências da Vida, Faculdade de Ciências e Tecnologia, Universidade NOVA de Lisboa, Caparica, Portugal

**Keywords:** nanoparticles, leukemia, lymphoma, diagnostics, therapeutics, nanotheranostics, nanomedicine

## Abstract

Nanotechnology has become a powerful approach to improve the way we diagnose and treat cancer. In particular, nanoparticles (NPs) possess unique features for enhanced sensitivity and selectivity for earlier detection of circulating cancer biomarkers. *In vivo*, NPs enhance the therapeutic efficacy of anticancer agents when compared with conventional chemotherapy, improving vectorization and delivery, and helping to overcome drug resistance. Nanomedicine has been mostly focused on solid cancers due to take advantage from the enhanced permeability and retention (EPR) effect experienced by tissues in the close vicinity of tumors, which enhance nanomedicine’s accumulation and, consequently, improve efficacy. Nanomedicines for leukemia and lymphoma, where EPR effect is not a factor, are addressed differently from solid tumors. Nevertheless, NPs have provided innovative approaches to simple and non-invasive methodologies for diagnosis and treatment in liquid tumors. In this review, we consider the state of the art on different types of nanoconstructs for the management of liquid tumors, from preclinical studies to clinical trials. We also discuss the advantages of nanoplatforms for theranostics and the central role played by NPs in this combined strategy.

## Introduction

Traditional approaches for cancer management rely on centralized diagnostic platforms that can be complex, time consuming and set for the wide spectrum of patients and malignancies (Friedman et al., 2015). These traditional approaches have been developed toward big data generation from pools of patients and for the identification of suitable biomarkers and profiles, which are the basis for standardization of chemotherapy protocols (Zaimy et al., 2017). Such strategies allowed for the development of precision oncology, in which profile data of the cancer cells enable a tailored treatment of individual patients, and have become a crucial trend for cancer management.

Nanoscale particles have been playing a central role in the detection of cancer biomarkers, which is essential for the personalized assessment at the basis of precision treatment. Versatile structural and functional properties of the nanoparticles (NPs) offer the possibility for rapid, specific, and sensitive diagnostics, toward decentralized assessment and/or ambulatory follow-up. In what therapeutics are concerned, the size range of these nanoconstructs allows them to cross biological barriers more effectively that may be further improved by functionalizing the nanoconstructs’ surface with specific ligands for precise delivery to the focus of disease (Lee et al., 2017). Moreover, NPs can work as therapeutic/imaging agents on their own or as carriers of multiple molecules that serve a specific function: tumor targeting, cancer cell ablation (*via* drug delivery or gene silencing), and real-time monitoring of cancer cells expansion or decay (Vinhas et al., 2015). This flexibility is crucial for theranostics, i.e., the simultaneous detection and therapy (Pedrosa et al., 2015). Most NP-based strategies have been directed at solid tumors, whereas the so-called non-solid tumors, such as lymphoma and leukemia, have not mobilized that much attention.

**Figure 1:**
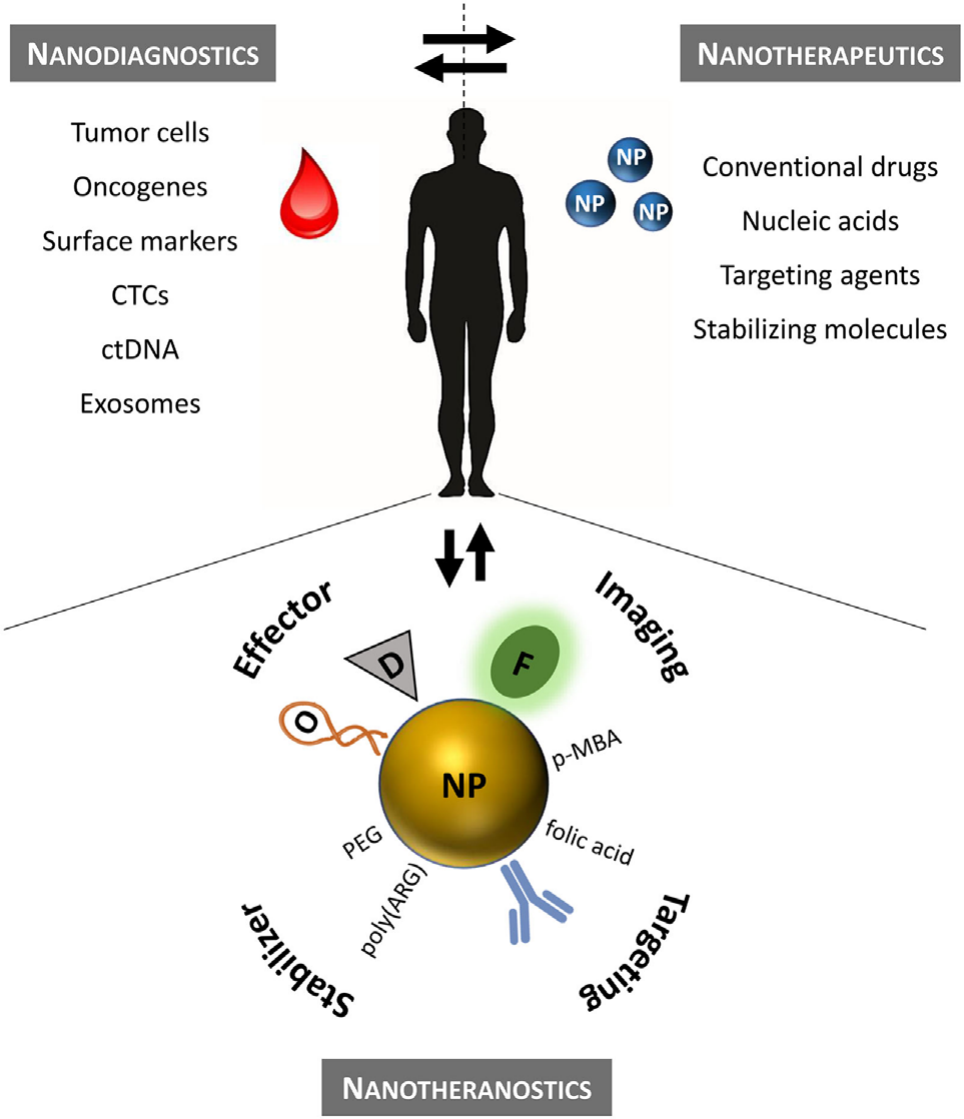
Precision nanomedicine for the management of hematological disorders. Nanodiagnostics based on liquid biopsies to assess multiple leukemia/lymphoma biomarkers using NP (*ex vivo* analysis). Nanotherapeutics according to the patient’s molecular profile (*in vivo*). Nanotheranostics combines diagnostics and therapeutics onto a single nanomaterial. Therapeutic efficacy may be improved for standard chemotherapeutics by efficient delivery to the target that can be monitored in real time. CTCs, circulating tumor cells; ctDNA, circulating tumor DNA; D, conventional drug; F, fluorophore; NP, nanoparticle; O, oligonucleotide; PEG, polyethylene glycol; p-MBA, p-mercaptobenzoic acid (Raman reporter); poly(ARG), poly-arginine peptide sequence.

Conventional chemotherapy in hematological cancers is challenged by the poor selectivity, resulting in low therapeutic efficacy and pronounced adverse side effects. These issues may be overturned by innovative nanomedicine approaches. In this review, we shall focus on nanotechnology advances that support leukemia and lymphoma biomarker detection and targeted treatment. Hematological disorders are very good candidates for such targeting since the molecular basis of each subgroup of patients is very well defined by common chromosomal translocations, shared mutations in oncogenes, gene expression profiles, and immune phenotype. Moreover, a simple blood sample (liquid biopsy) provides access to the patient’s full tumor profile, giving insightful information to support more focused therapeutic regimens—Figure 1. Unlike solid tumors that require NPs to reach the site of action, liquid tumors are spread throughout the bloodstream. Most of the barriers that NPs face to reach solid tumors are not critical in liquid tumors since circulating tumor cells are freely exposed to these agents. However, while in circulation, NPs may still be opsonized by blood proteins followed by recognition by the mononuclear phagocyte system (Sriraman et al., 2014). As such, liquid tumors require slightly different diagnostic treatment and targeting strategies that shall be herein discussed.

## Background of Liquid Tumors

Hematologic malignancies are the most common type of cancer among children and young adults, comprising leukemia, lymphoma, and myeloma, which affect the bone marrow, lymphatic system, and blood cells. The sequential stages of hematopoietic differentiation provide multiple opportunities for mutations to occur together with other disruptive events that lead to distinct tumor subtypes and clinical presentations (Hu and Shilatifard, 2016). The heterogeneity of tumors of the hematopoietic and lymphoid tissues presents unique challenges for current diagnosis and treatment (Den Boer et al., 2009; Tiacci et al., 2015; Cazzola, 2016; Freeman and Gribben, 2016).

Leukemia is a clonal disorder originated in the bone marrow during hematopoiesis and is characterized by the unregulated proliferation of poorly differentiated white blood cells. Classification of the disease is based on the type of cell affected (myeloid or lymphoid) and the degree of cell proliferation (acute or chronic) (Hu and Shilatifard, 2016). Acute myeloid leukemia (AML) is the most common type in adults (with an annual incidence of 4.2 cases per 100,000 persons and an annual mortality rate of 2.8 per 100,000) and acute lymphocytic leukemia (ALL) that is more prevalent among pediatric patients (with an annual incidence of 1.7 cases per 100,000 persons and an annual mortality rate of 0.4 per 100,000) (Siegel et al., 2016). Chronic myeloid leukemia (CML) is a myeloproliferative disorder with an annual incidence of 1.8 cases per 100,000 adults, accounting for 15–20% of newly diagnosed cases of leukemia in adults, and an annual mortality rate of 0.4 cases per 100,000 adults (Jabbour and Kantarjian, 2012; Siegel et al., 2016).

Lymphoma originates in lymph nodes where the lymphoid lineage of hematopoiesis differentiates into B-cells, T-cells, or natural killer cells. Abnormal events include extensive cell proliferation, somatic mutations, and antibody class switching, that ultimately impair the immune system as a whole and the adaptive immune response in particular (Young and Staudt, 2013). It is the most common hematologic cancer in western countries with an annual incidence of 19.5 cases per 100,000 persons. The number of deaths is circa 5.9 per 100,000 per year, with the majority being associated with aberrant B-cells, tackled using immunomodulating agents, monoclonal antibodies, or kinase inhibitors.

### Molecular Characterization

The foundational diagnosis of blood cancers is clinical, morphological, and immunophenotypic (usually *via* flow cytometry or immunocytochemistry) (Keeney et al., 2017), which is then followed by molecular characterization. Here, genetics is critical for the classification of these diseases and commonly assists risk stratification, from poor to favorable prognosis (Tasian and Hunger, 2017; Taylor et al., 2017). Treatment depends on the type of leukemia/lymphoma, disease stage, prior history of treatment, age, overall condition, and genetic profile. Most patients are treated with chemotherapy, and some may also undergo radiotherapy, stem cell transplantation, or targeted therapy (Zimmermann et al., 2016; Cuellar et al., 2017; Donato et al., 2017). CML is a good example of how hematological diseases have greatly benefited from the advance of cytogenetic and molecular methodologies. It was the first cancer in which a unique causative chromosomal abnormality was identified, t(9;22)(q34;q11)—Philadelphia chromosome (Ph)—and the associated *BCR-ABL1* gene, providing a specific target for disease treatment (Vinhas et al., 2017a,b). Tyrosine kinase inhibitors (TKIs) target the dysregulated kinase activity of the fusion protein encoded by *BCR-ABL1* (Campiotti et al., 2017; Patel et al., 2017).

### Liquid Biopsies in Leukemia and Lymphoma

The diagnosis and molecular management of cancer are mainly performed on biopsies, which allow histological and genetic characterization of the tumor—a valuable tool to characterize subtype and correlate to therapy protocol and, eventually, predict therapeutic response (Crowley et al., 2013; Brock et al., 2015). In solid tumors, biopsies correspond to a small portion of the neoplasm, not representing the pronounced heterogeneity. Usually performed at a specific time point, the dynamics of the tumor and variation of the mutation pattern are neither assessed nor represent the whole cancer. Taking multiple biopsies from patients is impractical, costly, whose invasiveness presents considerable risk and, above all, tremendous discomfort to the patient. In what myeloid or lymphoid neoplasms are concerned, conventional diagnostics consist of blood counts and flow cytometry markers evaluation directly on peripheral blood cells, which are also the tumor *itself* (Kumar et al., 2016).

From a peripheral blood sample, it is possible to obtain circulation tumor cells (CTCs), circulating cell free DNA, and other circulating biomarkers, such as microRNAs (miRs) and vesicles, which can provide valuable information before and during treatment in a non-invasive way (Brock et al., 2015; Buder et al., 2016; Krishnamurthy et al., 2017; Ranuncolo, 2017; Zhang et al., 2017). Liquid biopsies for solid or liquid tumors are associated with circulating material—mainly CTCs, nucleic acids, and exosomes. In liquid tumors, a direct sample of tumor cells may be retrieved from peripheral blood enabling the usual assessment of leukocytes, hemoglobin, lymphocytes, and platelet levels, together with prognostic markers, regarding disease outcome and clinical decision-making (Gomes et al., 2017).

## NPs Applications in Liquid Tumors

Cancer nanomedicine has raised the stakes in the detection of cancer biomarkers. Therapeutics could also benefit from nanotechnology-based strategies, which tend to be more selective than traditional chemotherapy. Among the plethora of nanoscale systems for cancer diagnostics and therapeutics, this review will focus on NPs. NPs are synthesized from diverse materials that, when at nanoscale, feature unique electronic, optical, and catalytic properties (Hasan, 2015; Nikalje, 2015; Tatar et al., 2016). There is a wide variety of NPs: organic, inorganic, and hybrid NPs. Due to their small size (optimal for intracellular uptake) and their large surface ratio (allowing functionalization with different biomolecules), NPs have been extensively used for diagnostic and therapeutic purposes (Tinkle et al., 2014; Venkatraman, 2014; Mendes et al., 2016). These nanosystems not only provide new prospects to overcome limitations associated with traditional drugs but also enable the incorporation of diagnostic and therapeutic functions onto a single platform, conveying nanotheranostics strategies for personalized medicine.

Organic NPs have been the most explored nanocarriers in cancer, including (i) dendrimers, which are highly branched, allowing the delivery of cargo and produced by controlled polymerization with a maximum of 10 nm of size; (ii) lipid-based NPs, such as liposomes and micelles that may carry a hydrophobic cargo and typically have 50–100 nm of size; and (iii) polymeric NPs, produced in a size range from 10 to 400 nm from synthetic, natural, hydrolytically, or enzymatically degradable polymers onto which a cytotoxic drug can be covalently attached, dissolved, encapsulated, or entrapped (Dawidczyk et al., 2014; Bhatia, 2016; Mendes et al., 2016). Organic NPs usually show high biocompatibility, whereas inorganic NPs provide advantages in terms of function and properties. The latter include carbon-based NPs (e.g., carbon nanotubes, buckyballs, and graphene), which feature remarkable strength and unique electrical properties (conducting, semi conducting, or insulating); metal NPs (e.g., gold, silver, and iron oxide); and quantum dots (QDs).

Among metal NPs, gold and ferromagnetic NPs have been explored the most. Gold NPs (AuNPs) are easy to synthesize and functionalize and have been used for drug and gene delivery, thermal ablation, and radiotherapy enhancement. AuNPs are also used in diagnostic applications due to their high sensitivity. Ferromagnetic NPs interest relies on their unique magnetic properties together with the possibility for surface functionalization, to be used as contrast agents for magnetic resonance imaging (MRI), hyperthermia, and drug delivery. QDs have been mainly explored for optical imaging since they show narrow and tunable emission spectra, a broad excitation spectrum and do not photobleach. QDs have also been proposed as therapeutic tool in conceptual *in vitro* and *in vivo* application. However, since they are synthesized from heavy metal elements, toxicity concerns have limited effective translation to the clinics.

### Nanodiagnostics in Liquid Tumors

Traditionally, leukemia and lymphoma cells are detected through morphological analysis, immunohistochemistry, antibody microarrays, flow cytometry using fluorescent markers, fluorescence *in situ* hybridization, PCR, and DNA sequencing. One of the major issues in lymphoid and myeloid neoplasms diagnostics is the limit of detection of immature white blood cells, since their numbers are very low at an early stage of the disease. Because these cancer types are extremely common and aggressive, effective treatment depends greatly on the accuracy and sensitivity of diagnosis. Signal amplification coupled with NPs may be a viable approach for earlier detection. For example, fluorescent QDs and nanometer semiconductors, with their superior fluorescent features, have been used for optimized detection schemes. In addition to fluorescent enhancement, metal NPs unique physicochemical properties, including localized surface plasmon resonance (LSPR), photoluminescence, or superparamagnetic properties, have also been explored (Sharma et al., 2015)—see Table 1.

**Table 1:** NPs-based diagnostic strategies for leukemia and lymphoma diseases.

Type of NP	Tumor	Target	Approach	Reference
Aptamer-conjugated Au-coated magnetic NPs	AML	PTK7	Aptamer (sgc8) recognizes PTK7 on screen-printed graphene–nitrogen nanosheet electrode (N-GN) *in vitro* cells and human blood plasma	Khoshfetrat and Mehrgardi (2017)
Hollow core photonic crystal silver NPs	AML	Leukemia cells	Portable monitoring of leukemic cells *in vitro*, using NPs for SERS	Khetani et al. (2015)
QDs-bsb-apt	ALL	PTK7	Aptamer (sgc8) for imaging tumor cells *in vitro* and *in vivo*.	Yu et al. (2016)
Aptamer-functionalized fluorescent silica NPs	ALL	PTK7	Sgc8-FSNPs specifically recognize leukemic cells *via* fluorescence *in vitro* and *in vivo*	Tan et al. (2016)
ssDNA-AuNPs	CML	e14a2 fusion transcript	Gold nanoprobes hybridize to *BCR-ABL1* sequence present in clinical samples (*ex vivo*) in colorimetric output	Vinhas et al. (2016)
Gold nanobeacons	CML	e13a2 and e14a2 fusion transcripts	FRET-based spectral codification for discrimination of the two most common *BCR-ABL* fusion transcripts *in vitro* leukemic cells	Cordeiro et al. (2016)
Anti-CD20-polymeric NPs	CLL	CD20	*In vivo* imaging Cy5.5-labeled-probe for CD20-expressing tumors based on fluorescence intensity	Capolla et al. (2015)
PEG-coated SERS AuNPs	Malignant B-cells (leukemia/lymphoma)	CD45, CD19, and CD20	Triplex spectra for SERS flow cytometry detecting anti-CD20 SERS probes in clinical samples (*ex vivo*)	MacLaughlin et al. (2013)
Anti-CD20 antibody-conjugated MNPs	Lymphoma cells	CD20	CTCs detection and isolation *in vitro via* MNPs functionalized with anti-CD20 antibodies	Sahoo et al. (2017)
QDs-labeled oligonucleotide probes	Acute leukemia and follicular lymphoma	Myeloperoxidase, bcl-2, survivin, and XIAP	Combination of oligonucleotide probes with spectral imaging for multiplex ISH in FFPE human tissue biopsies	Tholouli et al. (2006)

*AML, acute myeloid leukemia; ALL, acute lymphocytic leukemia; AuNP, gold nanoparticle; CML, chronic myeloid leukemia; CLL, chronic lymphocytic leukemia; PTK7, protein tyrosine kinase 7; FRET, fluorescence energy transfer; MNP, magnetic nanoparticles; NP, nanoparticle; PEG, polyethylene glycol; QD, quantum dot; SERS, surface-enhanced Raman scattering; ISH, in situ hybridization; FFPE, formalin-fixed paraffin embedded tissues; XIAP, X-linked inhibitor of apoptosis protein; CTC, circulation tumor cell*.

Aptamer-based nanodiagnostics systems have been proposed for acute leukemia *via* an antileukemia-thiolated aptamer (sgc8c) that specifically recognizes protein tyrosine kinase 7 (PTK7), an overexpressed transmembrane receptor in human T-cell ALL cells. Aptamers feature high affinity and selectivity toward their targets and have been used to functionalize AuNPs. These aptamer–NP systems have enhanced detection sensitivity, with a detection limit of 10 cells/mL, capable of differentiating leukemic from normal cells (Khoshfetrat and Mehrgardi, 2017). Yu et al. (2016) used aptamer-functionalized QDs to specifically detect leukemia cells in buffer and serum, whose toxicity was also evaluated in animal models, suggesting that the complex might be used both *in vitro* and *in vivo* (Table 1).

Chronic myeloid leukemia is associated with a unique chromosomal abnormality—*BCR-ABL1* gene—which is used for unambiguous molecular diagnostics. A review of the literature indicates that nanodiagnostics strategies for CML have relied solely on gold NPs (see Table 1). Based on the AuNPs’ LSPR, colorimetric detection of this molecular aberration has been achieved directly on total RNA purified from blood (Vinhas et al., 2016, 2017a). Cordeiro et al. (2016) used the AuNPs’ capability to modulate fluorescence emission of nearby fluorophores to selectively detect BCR-ABL1 isoforms. AuNPs functionalized with specific ssDNA oligonucleotides allowed the identification of *BCR-ABL1* positive samples, discriminating between the e14a2 and e13a2 fusion transcripts.

Most targets in leukemia and lymphoma diagnostics are antigens, such as CD20 that is overexpressed by malignant of B-cells (Tazi et al., 2011). CD20 antibody (rituximab) has been applied to the treatment of lymphoma but it can also be used for lymphoid neoplasms diagnosis. Sahoo et al. (2017) used avidin-modified magnetic NPs (MNPs) functionalized with biotinylated anti-CD20 antibody and a permanent magnet to specifically detect and isolate lymphoma cells from mixed samples. Capolla and colleagues also used an anti-CD20 antibody functionalized into polymeric-fluorophore Cy5.5-labeled NPs for *in vivo* imaging directed at the diagnosis of B-cell malignancies. These NPs might also be used as a personalized treatment strategy by loading with drugs, becoming an attractive theranostics platform (Table 1) (Capolla et al., 2015). Besides CD20 antigens, CD45 and CD19 are the two most common surface proteins expressed by B-cells and used in diagnostic immunophenotyping. MacLaughlin et al. (2013) reported the use of surface-enhanced Raman scattering (SERS) for detection of these three surface proteins in an *ex vivo* model of malignant B-cells. AuNPs were functionalized with one of the specific monoclonal antibodies (anti-CD20, anti-CD45, or anti-CD19) for SERS identification of target cells *via* flow cytometry. This strategy represents a significant step in the development of SERS immunophenotyping, improving sensitivity and specificity in blood cancer diagnosis (Table 1).

### NP-Based Strategies for Treatment of Liquid Tumors

Current treatment for leukemia and lymphoma involve chemotherapy and radiation, which often induce long-term side effects and multidrug resistance. In addition, other more invasive strategies that require a matching donor, such as stem cell transplant, have also been proposed. Nanotechnology provides the possibility to selectively deliver a high payload of anticancer agents to malignant cells without damaging healthy cells or systemic toxicity, allowing them to reach critical tissue compartments, such as the lymph nodes and the bone marrow, otherwise inaccessible to drugs—see Table 2.

**Table 2:** Recent studies (2015–2017) using nanoconjugates to improve leukemia and lymphoma management.

Nanoconjugate	Effector molecule	Targeting agent	Imaging agent	Condition (*in vitro*/*in vivo*)	Reference
QD-CdTe@Wogonin	Wogonin	n.a.	n.a.	*In vitro/in vivo* multidrug-resistant leukemia (K562-A02 cell line; leukemia-bearing mice)	Huang et al. (2016)
MNP-Fe_3_O_4_@Wogonin	Wogonin	n.a.	MNPs as contrast agents for MRI	*In vitro* multidrug-resistant leukemia (K562-A02 cell line)	Peng et al. (2016)
MNP-Fe_3_O_4_@SiO_2_@Cytarabine	Cytarabine	n.a.	MNPs as contrast agents for MRI	*In vitro* acute leukemia (HL60 and KG1 cell lines) Burkitt’s lymphoma (Raji cell line)	Shahabadi et al. (2016)
AuNP@BIRC5@Dasatinib or AuNP@BIRC5@Cy5 (controlled drug release *via* presence of *BIRC5* mRNA)	Dasatinib	*BIRC5* dsDNA oligonucleotide	Cy5	*In vitro/in vivo* CML (K562 cell line; K562-derived murine xenografts)	Gossai et al. (2014)
AuNP@FLT3-inhibitor	Lestaurtinibmidostaurin sorafenib quizartinib (FLT3 inhibitors)	n.a.	n.a.	*In vitro* AML (THP1 and OCI-AML3 cell lines)	Simon et al. (2015) and Petrushev et al. (2016)
AuNP@Fludarabine@Folic acid	Fludarabine phosphate	Folic acid	n.a.	*In vitro* AML (KG1 cell line)	Song et al. (2016)
AuNP@PEG@e14a2	BCR-ABL1 ssDNA oligonucleotide (e14a2)	n.a.	n.a.	*In vitro* CML (K562 cell line)	Vinhas et al. (2017b)
AgNP@p-MBA@Rituximab	Rituximab	Rituximab (detection of CD20)	p-Mercaptobenzoic acid (p-MBA) (Raman reporter and linker molecule)	*In vitro* Burkitt’s lymphoma (Daudi and Raji cell lines)	Yao et al. (2016)
SLN@ATRA (cholesteryl butyrate-solid LNPs, SLN)	All-trans retinoic acid (ATRA)	n.a.	n.a.	*In vitro* acute leukemia (HL60, Jurkat and THP1 cell lines)	Silva et al. (2016)
LNP@Mcl1	*Mcl1* siRNA	n.a.	n.a.	*In vitro* mantle cell lymphoma [JeKo-1 (normal) and MAVER-1 (aggressive) cell lines]	Knapp et al. (2016)
LNP@antagomiR126@Anti-CD45.2 (lipopolyplex NPs)	AntagomiR-126	CD45.2 antibody	n.a.	*In vitro/in vivo* AML (human primary cells; murine model)	Dorrance et al. (2015)
T-cells@LNC@SN-38@PEG [lipid nanocapsule (LNC)]	Topoisomerase I poison SN-38	Healthy primary T-cells (live vector)	n.a.	*In vivo* lymphoma (murine model)	Huang et al. (2015)
Nanopolymer-PTL@pSi@ESTA [micellar NPs protected by porous silicon (pSi) coating]	Parthenolide (PTL)	E-selectin thioaptamer (ESTA) (bone marrow-directed aptamer)	n.a.	*In vivo* AML (patient-derived murine xenografts)	Zong et al. (2016)
Nanopolymer@DOX@Anti-CD19	DOX	CD19 antibody	n.a.	*In vitro/in vivo* ALL (REH and RS4; 11 cell lines: human-derived murine xenografts)	Krishnan et al. (2015)
MSN@DOX@PEG@Rituximab (pH-controlled delivery *via* MSNs)	DOX and rituximab	Rituximab (CD20 antibody)	n.a.	*In vitro/in vivo* B-cell lymphoma (Raji and Daudi cell lines; murine model)	Zhou et al. (2017)
DNP@BCR-FITC@polyArg-BCL2 [diatomite NPs (DNPs)]	*BCL2* siRNA	Idiotype-peptide specific BCR	FITC	*In vitro* lymphoma (murine A20 cell line)	Martucci et al. (2016)
Rituximab-ABX-Alexa750 (rituximab binds to albumin of ABX, NP-albumin-bound PTX)	PTX and rituximab	Rituximab (CD20 antibody)	AF750	*In vitro/in vivo* B-cell lymphoma (Daudi cells; human B-cell lymphoma murine model)	Nevala et al. (2017)

*AF750, alexafluor 750; AML, acute myeloid leukemia; CML, chronic myeloid leukemia; DOX, doxorubicin; n.a., not applicable; PEG, polyethylene glycol; polyArg, peptide sequence with nine arginines; PTX, paclitaxel; NP, nanoparticle; ALL, acute lymphocytic leukemia; QD, quantum dot; MNP, magnetic NP; BCR, B-cell receptor; LNP, lipid NP; siRNA, small interfering RNA; MSN, mesoporous silica NP; ABX, ambraxane*.

#### Quantum Dots

Quantum dots as nanocarriers have also been used to enhance antitumoral drug efficacy. Recently, 4 nm diameter cadmium-telluride QDs were conjugated with wogonin, a natural flavonoid with antiproliferative activity against several cancers, to induce apoptosis and differentiation of tumor cells (Xu et al., 2014; Yang et al., 2014; Hu et al., 2015). These nanocomposites overcame multidrug-resistant leukemia by facilitating interaction between wogonin and abnormal cells (Huang et al., 2016).

#### Metal NPs

Numerous studies examine the robustness of metal NPs to potentiate current cancer therapy. Above all, AuNPs and MNPs exhibit good biocompatibility, low toxicity, biodegradability, and high volume-to-surface ratio. MNPs can be directed toward the tumor site using a magnetic field and are very good contrast agents in MRI (Estelrich et al., 2015; Gobbo et al., 2015). MNPs ranging from 12 to 23 nm have been combined with wogonin or cytarabine (mainly used to treat AML) to selectively tackle multidrug-resistant leukemia cells *in vitro*, acute leukemia, and Burkitt’s lymphoma (Peng et al., 2016; Shahabadi et al., 2016).

AuNPs are highly customizable to deliver a drug specifically to a particular cell or tissue cell. In 2016, Gossai et al. functionalized 15 nm AuNPs with dsDNA oligonucleotides with a sequence corresponding to *BIRC5*, a gene is overexpressed in CML cell lines, which was further loaded with dasatinib, a potent TKI frequently used against CML. Inside the cells, the target gene mRNA binds to the antisense oligonucleotide and releases the drug-conjugated DNA oligonucleotide proportionally to level of *BIRC5* mRNA in those cells (Gossai et al., 2014). Other studies examined the *in vitro* efficacy of drug-coated AuNPs on AML treatment improvement using TKIs and fludarabine (Simon et al., 2015; Petrushev et al., 2016; Song et al., 2016). One of these systems took additional advantage of folate grafted to the NPs’ surface. Because folate receptors are highly overexpressed on the surface of tumor cells these nanoconjugates were able to actively target AML cells (Song et al., 2016).

AuNPs provide protection against degradation by RNases, thus increasing circulating times and subsequent increase of the payload of drug delivered to cells. A recent study conjugated 14 nm AuNPs with hairpin ssDNA oligonucleotides that recognize the fusion transcript *BCR-ABL1*, the molecular hallmark in CML to tackle this cancer (Suka et al., 2016). This strategy resulted in *BCR-ABL1* silencing and significant CML cell death, with high selectivity since it had no effect on cells that do not express the *BCR-ABL1* transcript. Recently, Vinhas et al. (2017a,b) combined the silencing potential of oligonucleotide functionalized AuNPs with imatinib. Combination of these agents selectively decreased cell survival, inducing loss of viability of imatinib-resistant K562 cells, showing great promise to overcome TKI resistance in Ph+ diseases.

#### Lipid NPs (LNPs)

Lipid NPs show plenty of applications for drug delivery since they improve the stability of pharmaceuticals, be it lipophilic or hydrophilic molecules (Miao et al., 2017). LNPs have been used as vehicles for antileukemia all-trans retinoic acid (Silva et al., 2016), small interfering RNA (siRNA) technology to silence *Mcl-1* expression in mantle cell lymphoma *in vitro* models (Knapp et al., 2016), and target miRs overexpression in leukemic stem cells believed to be the cause of AML chemoresistance and relapse (Dorrance et al., 2015). Huang and colleagues used LNPs to actively target the potent topoisomerase I poison SN-38. Healthy T-cells were engineered to retain homing receptors that mirror lymphoma cell biodistribution and to be resistant to SN-38. These live vectors were then used to carry lipid nanocapsules (LNCs) conjugated to SN-38 (LNC@SN-38; 136 nm diameter) to reduce tumor burden and improve survival in murine models after only 2 weeks of treatment. Conveyed by these nanocapsules, SN-38 levels in lymph nodes were almost 1,000× higher than for the free SN-38 (Huang et al., 2015).

#### Polymeric NPs

Polymeric NPs, typically formed through the assembly of copolymers, have been used to deliver a spectrum of chemotherapeutic, diagnostic, and imaging agents in cancer (Kallinteri and Garnett, 2007). In one such examples, parthenolide (PTL), a preclinical agent that can eliminate resistant AML stem cells while preserving normal hematopoietic function, was first incorporated into mPEG-polylactic acid micelles, encapsulated in a protective degradable porous silicon (pSi), and coated with E-selectin thioaptamer (ESTA) to direct the particles toward the bone marrow (Zong et al., 2016). ESTA binds to the adhesion molecule E-selectin expressed exclusively by bone marrow endothelial cells (Winkler et al., 2012). The developed multistage vector successfully delivered PTL to the bone marrow of patient-derived AML xenografts. Two doses of the nanoconjugate, administered with a 2-week interval, were enough to impair leukemic stem cell function and AML burden *in vivo* (Zong et al., 2016).

Co-polymeric NPs were also used to deliver anticancer drug doxorubicin (DOX) against ALL. These DOX-coated NPs were further functionalized with CD19 antibodies to enhance their internalization *via* receptor-mediated endocytosis in CD19 positive ALL cells with minimal cytotoxicity toward healthy cells. Indeed, the 83 nm nanoconjugates were tested in ALL mice, exhibiting higher therapeutic efficacy and reduced systemic toxicity than that of free DOX (Krishnan et al., 2015).

#### Mesoporous Silica NPs (MSNs)

Mesoporous silica NPs are also suitable for nanotherapeutics in cancer due to their unique porous structure, tunable pore size, and the ability to release drugs in response to pH, temperature, light, redox reactions, enzymes, or biomolecules (Cheng et al., 2017; Lin et al., 2017; Yan et al., 2017; Zhan et al., 2017). Zhou et al. (2017) incorporated DOX into the pores of pH-sensitive MSNs, whose pH-responsive mechanism enabled the controlled release of low levels of drug at a physiological pH and efficient intracellular release under more acidic conditions, characteristic of endosomal and lysosomal environments. To improve targeting of MSN@DOX to tumor cells, these NPs were functionalized with rituximab *via* an avidin–biotin system. *In vitro* tests showed that the targeted system was internalized more efficiently by CD20 positive than by CD20 negative cells. *In vivo* tests confirmed the effective deliver of DOX to lymphoma B-cells, inducing cell apoptosis and inhibiting tumor growth with minimal toxic side effects.

## Future Perspective on Nanotechnology for Hematological Diseases

### Nanotheranostics

Many features of NPs herein described are particularly attractive for biomarker detection and simultaneous abnormal cells ablation, making them suitable candidates for nanotheranostics (Chen et al., 2017). In liquid cancers, tumor cells are free in circulation, requiring specific active targeting. However, confined tumor sites are also present, such as the bone marrow and/or lymphoid tissues, nanosystems profiting from the EPR effect may be of extreme relevance in tackling these niches. This way, therapeutic nanoconjugates may accumulate at tumor locations, where subsequent active targeting to cancer cells may be achieved (Lammers et al., 2012). A few preclinical studies on lymphoma nanotheranostics using metal NPs, diatomite NPs (DNPs), and nanoantibodies (nanobodies) have already been reported—see Table 2.

For example, 50 nm AgNPs have been conjugated to p-mercaptobenzoic acid—a Raman reporter and linker molecule, together with rituximab, for B-cell lymphoma targeting and ablation—to allow non-invasive detection of living cells without labeling based on SERS. In addition, these silver nanostructures were tuned to enhance SERS signals from single molecule, thus allowing multiplexing. The so designed Ag nanoconstruct was capable to simultaneously detect CD20 positive single lymphoma cell and eliminate them with high selectivity (Yao et al., 2016).

Diatomite NPs, silica-based NPs of irregular shape and mean size of approximately 200 nm, were also applied in the management of B-cell lymphoma (Martucci et al., 2016). These DNPs have been modified to actively target the hypervariable region of surface immunoglobulin B-cell receptor (BCR) toward fluorescence-based monitorization *via* FITC and confocal microscopy/flow cytometry. These conjugates also simultaneously recognize and downregulate the antiapoptotic factor B-cell lymphoma/leukemia 2 (Bcl2) mediated by siRNA therein encapsulated. The nanostructure successfully mediated *in vitro BCL2* gene silencing in a target selective manner. This approach could be applied in the follow-up of lymphoma/leukemia patients.

Another example of nanotheranostics using a nano-antibody composed of rituximab conjugated to an NP albumin-bound paclitaxel [ambraxane (ABX)] has been developed toward decimate B-cell lymphoma (Nevala et al., 2017). *In vivo* imaging of the tumor burden reduction was achieved by labeling the ABX with alexafluor 750. The 160 nm nanocontruct retained the cytotoxicity of ABX and the CD20 affinity of rituximab, both *in vitro* and *in vivo*. Moreover, by combining both antibodies at nanoscale, higher therapeutic efficacy was achieved when compared with ABX or rituximab alone.

### Current Limitations of NPs and Leukemia/Lymphoma *In Vivo* Models

Thus far, nanoformulations have enhanced biomarker detection, providing simpler assays with higher sensitivity. Nanomedicines have also been shown to improve the efficacy–toxicity ratio of anticancer agents, thus offering the possibility to monitor diagnostics and treatment of liquid tumors in real time. However, the success of nanomedicines at a preclinical stage depends greatly on the availability of *in vivo* tumor models that mimic the real human tumor environment. Leukemia/lymphoma models show several obstacles since the pathogenesis of the disease in murine models is not relevant to most human cases; also, these models fail to replicate the complex microenvironment from which these human cancers arise, and do not embody their genetic and molecular heterogeneity (Cook and Pardee, 2013; Kohnken et al., 2017). Xenografts mitigate some of these issues, but they are usually conducted on immunocompromised mice to avoid immune rejection of human cells, which excludes the effects of the immune system on tumor expansion and on NPs efficacy and targeting.

The variability of experimental conditions between different preclinical studies using NPs to tackle leukemia and lymphoma also contribute to their reduced clinical impact. There is a lack of standardized manufacturing procedures and controls, recognized by regulatory agencies—US Food and Drug Administration (FDA) or the European Medicines Agency—for clinical translation of nanoscale diagnostic assays and treatment. In addition, there have been a deficiency on *in vivo* toxicity, stability, and biodistribution studies that are essential to determine NPs ability as delivery vehicle, imaging, or therapeutic agent (Dawidczyk et al., 2014; Shi et al., 2016).

### Clinical Trials

Preclinical studies of nanomedicines against hematological malignancies hold great promise but thus far only but a few have reached the clinical trial stage. Of these, liposomal nanoformulations show some impact in blood malignancies, and three liposomal formulations for the treatment of leukemia have reached advanced clinical trials. In 2012, liposomal vincristine sulfate (Marqibo^®^) was the first nanoformulation to get approval by the FDA to treat Ph+ ALL in adults that relapse or that do not respond to at least two antileukemia drugs. Vincristine inhibits microtubule formation in mitotic spindle, resulting in an arrest of dividing tumor cells at the metaphase stage and is a standard component of chemotherapy regimens used to treat ALL and other lymphoid malignancies for over 50 years. However, vincristine’s free version induces major side effects. Encapsulation in a sphingomyelin/cholesterol-based liposome changed vincristine pharmacokinetics dramatically, leading to a slow release from vector and delivery to tissues more efficiently, allowing administration of higher doses (ClinicalTrials.gov Identifier: NCT01439347) (Douer, 2016). Marqibo^®^ has also completed phase I clinical trials for the treatment of pediatric ALL (ClinicalTrials.gov Identifier NCT01222780) (Shah et al., 2016).

Other examples of promising nanomedicines currently in advanced stage of development include the following: (i) CPX-351, a liposomal formulation of cytarabine and daunorubicin, that yielded interesting results in phase III clinical trials for the treatment of high-risk AML. In fact, CPX-351 significantly improved overall survival, event-free survival, and response rates in comparison with the standard regimen of cytarabine and daunorubicin (ClinicalTrials.gov Identifier: NCT02286726); (ii) annamycin, an anthracycline intended for the treatment of relapsed or refractory leukemia, has also been encapsulated in a liposome and submitted to phase I/II multicenter clinical trials. The drug was well tolerated and showed encouraging antileukemic activities against ALL (ClinicalTrials.gov Identifier: NCT00271063) (Wetzler et al., 2013).

Future studies have been proposed focusing on the combination of these nanoformulations with other antileukemic drugs, namely, TKIs, and incorporate new classes of therapeutic agents, such as, siRNA, miRs, ssDNA, and gene editing.

## Conclusion

Cancer nanomedicine has been evolving steadily for the past 10–15 years, putting forward solutions for more accurate diagnostics, including molecular characterization in rapid point-of-need platforms, and radical new approaches of smart NPs for therapy. However, these advances have yet to prove efficacy in the clinics, before eventually becoming routine substitutes of traditional chemo/radio therapy. The vast majority of these nanomedicines have focused on tackling solid tumors, with the characteristic implantation, invasion, inflammatory response, and metastasis. Liquid tumors have been somehow neglected, but new strategies for both diagnostics and therapeutics are slowly gaining pace. Nanomedicine holds great promise to tackle liquid tumors, both at the local environment (e.g., bone marrow) and in circulation since most malignant cells are spread throughout the body *via* the blood and lymph. Newer and smarter NP-based approaches are required to eliminate these cancer cells with increased efficacy and specificity.

## Author Contributions

PB and AF outlined the project. All the authors reviewed the literature, drafted the manuscript, and approved it for publication.

## Conflict of Interest Statement

The authors declare that the research was conducted in the absence of any commercial or financial relationships that could be construed as a potential conflict of interest. The reviewer CM and the handling editor declared their shared affiliation.
